# Demographics and access to head and neck cancer care in rural areas compared to urban areas in Germany

**DOI:** 10.1002/cam4.6505

**Published:** 2023-09-14

**Authors:** Julius M. Vahl, Gabriele Nagel, Ayla Grages, Matthias Brand, Adrian von Witzleben, Michael Sonntag, Marie‐Nicole Theodoraki, Jens Greve, Tsima Aboukors, Michael Denkinger, Dhayana Dallmeier, Christian Idel, Thomas K. Hoffmann, Simon Laban

**Affiliations:** ^1^ Department of Otorhinolaryngology and Head & Neck Surgery, Head and Neck Cancer Center of the Comprehensive Cancer Center Ulm University Medical Center Ulm Ulm Germany; ^2^ Department of Epidemiology and Medical Biometry University Medical Center Ulm Ulm Germany; ^3^ Agaplesion Bethesda Ulm, Geriatric Research Ulm University and Geriatric Center Ulm Germany; ^4^ Department of Otorhinolaryngology University Hospital Schleswig‐Holstein Lübeck Germany

**Keywords:** demographic change, East Germany, industrial country, peripheral region

## Abstract

**Background:**

Demographic development in rural and urban areas differs substantially. Demographics and access to specialized head and neck cancer centers may affect head and neck cancer patients' (HNCP) outcomes. Here, we compare epidemiological indicators and outcomes of HNCP in rural and urban Germany.

**Patients and Methods:**

In a retrospective analysis of data from the Center for Cancer Registry Data (ZfKD) between 2002 and 2017, 212,920 HNCP were included. Incidence, demographics, travel distance to specialized centers, and ground values were compared between rural and urban areas with a focus on their association with patient outcomes.

**Results:**

The mean age of HNCP was significantly higher in urban areas (mean difference = 1.4 years; *p* < 0.0001), but increased at a comparable rate (*p* = 0.26) in rural and urban areas during the observation period. Gender imbalance was higher in rural areas (mean ratio of men/women: 4.1 vs. 3.1; *p* < 0.0001), but showed a comparable trend toward equilibration in both, rural and urban districts (*p* = 0.46). The portion of HNCP of the entire HNCP population living in urban areas increased from 55.9% in the year 2002 to 76.4% in the year 2017. There was no significant difference or change in the ratio of advanced to low UICC stage during the observation period (*p* = 0.26). However, travel distances to medical centers were higher in rural areas, especially (*p* < 0.0001) in East Germany. Median survival of HNCP in rural areas was significantly lower than in urban areas (42 months [SEM = 0.7; CI: 40.5–43.5] vs. 54 months [SEM = 1.2; CI: 51.7–56.3]; *p* < 0.0001) in East Germany, whereas in West Germany no significant difference was observed (59 months [SEM = 0.8; CI: 57.4–60.6] vs. 60 months [SEM = 0.5; CI: 59.0–61.0]; *p* = 0.15).

**Conclusions:**

Place of residence contributes to survival outcome of HNCP. Access to specialized care and socioeconomic factors could be improved in East Germany.

## Graphical abstract

In a retrospective analysis of data from the German Center for Cancer Registry Data between 2002 and 2017, 212,920 head and neck cancer patients (HNCP) were included. The median survival of HNCP in rural areas was significantly lower than in urban areas (42 months vs. 54 months; *p* < 0.0001) in East Germany, whereas in West Germany no significant difference was observed (59 months vs. 60 months; *p* = 0.15). Access to specialized care and socioeconomic features seem to be crucial prognostic factors.

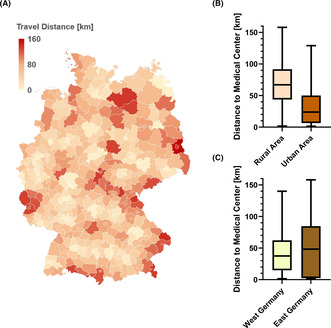

## 1 BACKGROUND

Germany is embedded in central Europe, bordered by nine nations and the North and Baltic Sea. Nowadays, it is an internationally respected economic driver in the Eurozone. As a result of the Second World War, Germany was divided into two states, which were reunited in 1990. Since 1991, Berlin has been the capital of the state made up of 16 federal states. Society and economy of Germany developed differently in both former states. Demographic changes also differ by its geography. Nevertheless, making distinctions between East and West or federal states alone is often inadequate when evaluating regional health differences.^1^ General life expectancy is known to be low in rural and urban regions with social deprivation (differences up to 5 years).^1^ Besides, the possibility of treatment at a clinical center has decisive advantages.^2^ Balancing quality of health care guidelines, also from the point of view of cost–benefit analysis, are frequently announced by the Institute for Quality and Efficiency in Health Care: Germany (IGWiG).^3^

Rural and urban areas are differently affected by demographic change. To assess spatial development, federal districts in Germany are distinguished by the German federal Institute for building, urban affairs and spatial development into rural and urban environments based on two main items: settlement density and geographical position.^4^

Spatial development affects many health‐related issues and has an impact on head and neck cancer (HNC). In Egypt, a developing country, HNC incidence was twofold higher in rural areas than in urban areas between 1999 and 2006.^5^ Furthermore, an examination of the American National Cancer Database (*n* = 146,256) revealed crucial survival disparities at the expense of rural HNC patients (HNCP) between 2004 and 2015.^6^ On the other hand, there are reports from England were no differences in survival outcomes between rural and urban areas were observed.^7^ Nevertheless, cancer registry data analysis (*n* = 817,182) of selected cancer types (not including HNC) in part of Germany (11 out of 16 federal states) between 1997 and 2006 did not reveal major outcome disparities between rural and urban areas.^8^ However, we recently published analyses of age and travel burden at the German tertiary cancer center Ulm^9^, ^10^ and also focused on the impact of demographic factors on survival of HNCP in entire Germany.^11^ Here we observed an increase in annual incidence rates, especially for HNCP being older than 70 years and a concomitant rise in the HNCP population's mean age over time (2004–2018).^9^ But in opposite to observations from overseas, no association of travel distance and TNM status or UICC stage at initial diagnosis has been detected. Furthermore, travel distance has not had any significant impact on recurrence‐free and overall survival.^10^

The main objective of just this study was to compare demographic development between rural and urban areas in Germany and their potential association with patients' outcome. We hypothesized that age, gender distribution, travel distances to specialized medical care centers, and socioeconomic factors summarized by the area mean land value differ between rural and urban areas potentially resulting in an outcome disparity in HNCP.

## 2 PATIENTS AND METHODS

### 2.1 Data collection

We requested current data of the German Center of Cancer of newly diagnosed patients with HNC for the time period from January 2002 to December 2017.^12^ We received anonymized data of 212,920 HNCP including gender, district of residence, anonymized date of birth and date of diagnosis, TNM (tumor, nodal, and metastasis) status, as well as survival follow‐up data (East Germany: December 2015; West Germany: December 2017). Furthermore, we requested data concerning the general German population between 2002 and 2017 at the central information service of the State Statistical Office Baden‐Wuerttemberg, which coordinated data retrieval from the 16 individual states and their statistical offices. Socioeconomic, publicly available data were enclosed in the analysis using the internet portal of the German statistical offices at November 20, 2022. Data on the distribution of various physicians and hospital capacities in Germany were obtained from publicly available download sections at the internet portal of national association of statutory health insurance physicians (KBV) on November 15, 2022.

### 2.2 Data exclusion

For the analysis of HNC incidence, TNM status, and demographics of HNCP we were able to use the major part of the dataset (*n* = 212,920). However, some data of TNM status were missing (Table 1). For 45.1% of HNCP no UICC stage (eighth edition) could be determined, because of missing data for TNM features. Notably, the federal state of Baden‐Württemberg started reporting data to the cancer registry in 2009 after it became obligatory due to legal requirements (Federal Cancer Registry Data Law; August 18, 2009). Also, a delay in reporting occurred in 2016 and 2017 resulting in incomplete data after December 2015 in some states. For example, no HNC reports were received from East Germany in 2017. Therefore, caution must be paid to the results of mean age, gender, UICC status, and residency development of HNCP during the years 2016 and 2017.

**TABLE 1:** TNM classification by residence.

HNCP		Rural area		Urban area	
*n* = 212,920		Total number	Percent	Total number	Percent
T	T1	16,583	26.3	30,306	30.1
	T2	15,523	24.6	26,709	26.5
	T3	10,959	17.4	17,610	17.5
	T4	15,266	24.2	20,724	20.6
	Tx	4821	7.6	5309	5.3
N	N0	23,313	42.2	38,049	43.3
	N1	7175	13.0	12,581	14.3
	N2	22,287	40.4	33,925	38.6
	N3	2418	4.4	3240	3.7
M	M0	48,117	94.3	68,626	94.1
	M1	2900	5.7	4299	5.9
Missing	T/N/M	24,747	33.8	71,213	51.0

*Note*: Tumor, nodal, and metastases status is illustrated in the table depending on residence.

Furthermore, to conduct the survival analysis, HNCP with missing survival data were excluded. This exclusion included patients with the initial diagnosis after 2016 in East Germany. HNCP reported as *dead* without a date of death, or *death certificate only* (*DCO*) cases and HNCP reported as *alive* without a date of latest follow‐up were excluded for survival analysis. Therefore, the cohort was filtered to *n* = 193,877 HNCP available for survival analysis.

### 2.3 Classification of rural and urban areas

The assignment of areas was obtained from the German federal Institute for building, urban affairs and spatial development based particularly on settlement density and geographical position. The differentiation of central or peripheral location is set on the basis of accessibility analyses of the relationship of residents and their workplaces.^4^ Further density‐related division of areas into four types can be performed considering settlement density and the fraction of cities in the area. Urban areas may be subdivided into big cities (>100,000 inhabitants/km^2^) and urban districts with at least 150,000 inhabitants per km^2^. Rural areas can accordingly be divided into rural districts with either high or low settlement density depending also on the fraction of cities. Second, based on geographical position, the location of an area may also be classified choosing one of four features: very peripheral, peripheral, central, and very central. This classification is based on motorized travel time to the individual workplace including commuters or rather the “daily population.” The application of a certain distance function weights shorter distances higher than more distant ones, because intensity of contact and interconnection among commuters is halved with increasing travel time approximately every 10–15 min. The assignment of areas to the settlement‐related spatial types is reviewed annually and is adjusted to the threshold values if a permanent undershooting or overshooting of the threshold values is assumed.^4^ A technical application of this classification can be found in the professional driver qualification law.^13^

### 2.4 Data arrangement

The Center for Cancer Registry delivered data summarized in an excel file. We imported these data to IBM SPSS Statistics 26 and GraphPad Prism 9 for statistical testing. Figures were arranged in GraphPad Prism and Microsoft Power Point 2019 and tables were created in Microsoft Excel 2019.

### 2.5 Calculation of travel distances

Due to data protection laws, we received anonymized residence data. Therefore, estimated mean travel distances to specialized care were calculated from the center point of the respective district to the nearest university hospital (38 in total in 2022), in kilometers using Google maps. University hospitals were used as a proxy for specialized head and neck cancer centers (HNCC). The certification of such centers has been established by the German cancer society in 2010. Distances were assigned to HNCP from the respective districts. Therefore, travel distance estimates can only provide an estimation of the actual individual travel distance assuming that patients choose the shortest route despite a free choice of treatment center in Germany.

### 2.6 Estimation of survival

Survival was calculated using the Kaplan–Meier method. Median overall survival of all patients was computed after application of above‐mentioned exclusion criteria with SEM (standard error of the mean) and 95%‐confidence interval (CI). The time frame of observation was limited to 10 years, which was useful to compensate for data collection errors in East Germany in the context of comparability between East and West.

### 2.7 Statistical analysis

To test whether mean values among two groups were significantly different, we first tested for normality distribution using the Shapiro–Wilk test (*n* ≤ 50) or Kolmogorov–Smirnov test (*n* > 50) and homoscedasticity by Levene's test. Then, we performed an unpaired t‐test if normal distribution and homoscedasticity were present. In cases of missing normality, we used nonparametric Mann–Whitney *U* test to compare scaled values among two groups. To test differences between groups with nominal and ordinal variables we used Chi square test (minimal requirements for sample size were met). Standard deviations are abbreviated with “SD.” Differences of slopes, assuming a linear relationship, were tested with Deming regression analysis. Deviation of the slope coefficient from zero was then tested by *F* test. Further correlation analysis between two groups was in our case done using Spearman estimation (r). Differences in survival data were tested with log rank test. When performing multivariate analysis with Cox regression, we first checked model fitting by Omnibus tests, then we analyzed hazard ratios (B), 95%‐confidence interval, level of significance, and correlation matrix of regression coefficients.

## 3 RESULTS

### 3.1 Patients' characteristics

**FIGURE 1:**
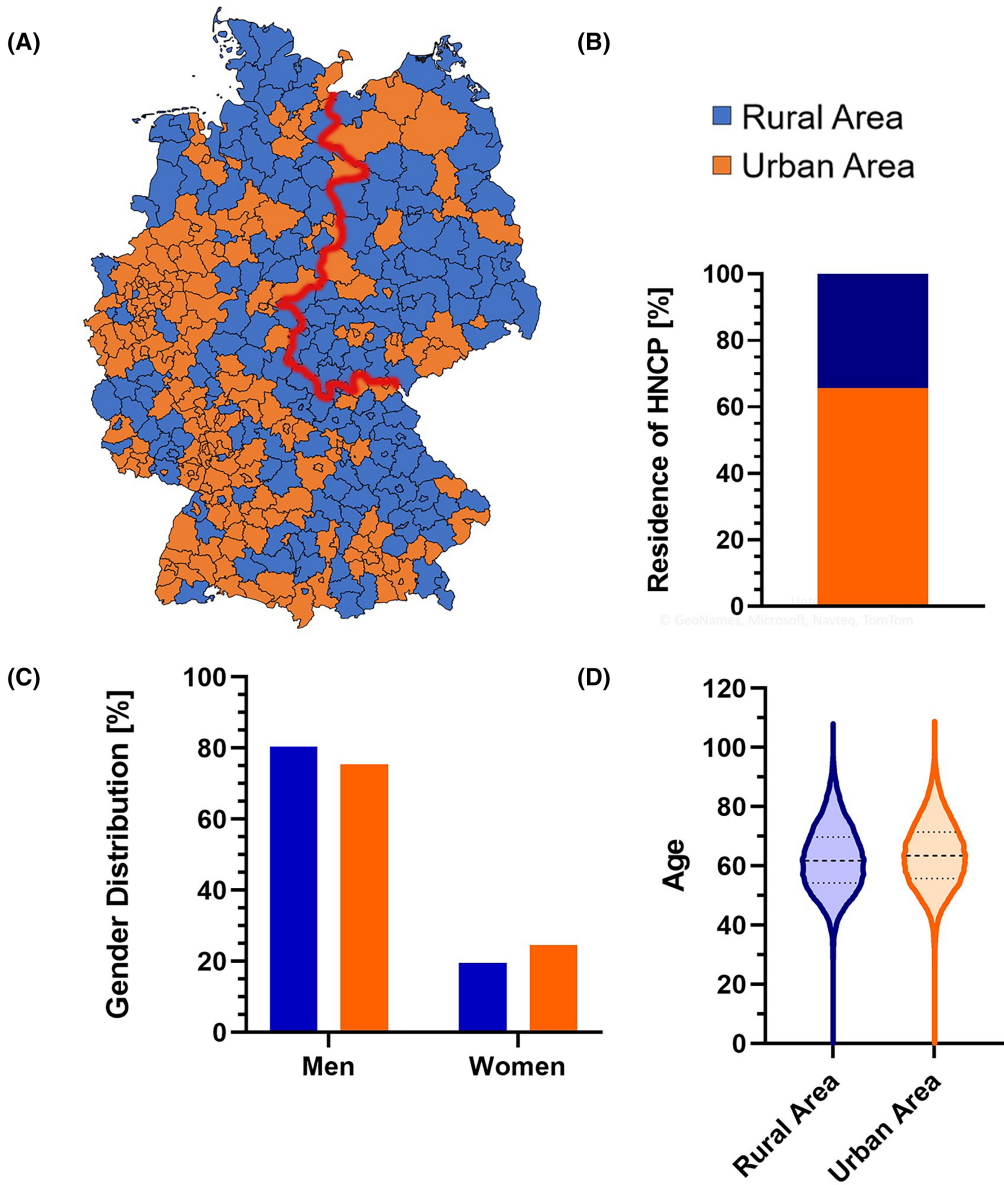
Illustration and characterization of rural and urban districts in Germany. (A) Visualization of rural and urban districts according to §22 of the professional driver qualification law based on data of the German federal institute for research on building, urban affairs (31.12.2017) with addition of the imaginary border between East and West Germany (red). (B) Bar graph of the relative distribution of head and neck cancer patients (HNCP) to rural and urban areas. (C) Relative HNCP gender distribution compared by rural and urban areas. (D) Median age with interquartile range and minimum/maximum for rural areas and urban areas respectively is shown.

In total, 212,920 HNCP were included in this analysis of German cancer registry data over 15 years from 2002 to 2017. About 34.4% of these reside in rural and 65.6% in urban areas (Figure 1A,B). The five districts with the highest numbers of HNC reports (Figure S1A) also belong to the ones with the highest population counts (Berlin, Hamburg, district of Hannover, Munich, Cologne). The gender distribution (Figure 1C) also differs between rural and urban area with a more pronounced underrepresentation of female HNCP in rural areas (urban area: 75.4% men and 24.6% women; rural area: 80.4% men and 19.6% women; *p* < 0.0001). The mean age of HNCP (Figure 1D) living in rural areas was significantly lower (62.2 years; SD = 11.3) than in urban areas (63.7 years; SD = 11.4; *p* < 0.0001). The composition of HNC primary sites among both groups (Figure S1B) also showed significant (*p* < 0.0001), but small differences. In rural areas 0.15% more oropharyngeal, 0.74% oral and 2.27% hypopharyngeal tumors were detected. On the other hand, 0.92% less laryngeal, 0.31% nasopharyngeal, and 1.94% other tumors were registered. In the same manner, stage distribution was analyzed (Figure S1C) and showed significant, but little differences (*p* < 0.001). In rural areas 0.87% less UICC I, 0.01% UICC II, and 0.85% UICC III, but 1.73% more UICC IV is observed. However, attention is warranted due to a high prevalence of missing data. In fact, for 45.1% of HNCP were missing at least one component of TNM status to compose UICC stage (East Germany: 24.9%; West Germany: 51.3%). Known TNM information is displayed separately for rural and urban areas including a section with missing data on at least one parameter of TNM status (Table 1).

### 3.2 Mean estimated travel distances are higher in rural areas, especially in East Germany

**FIGURE 2:**
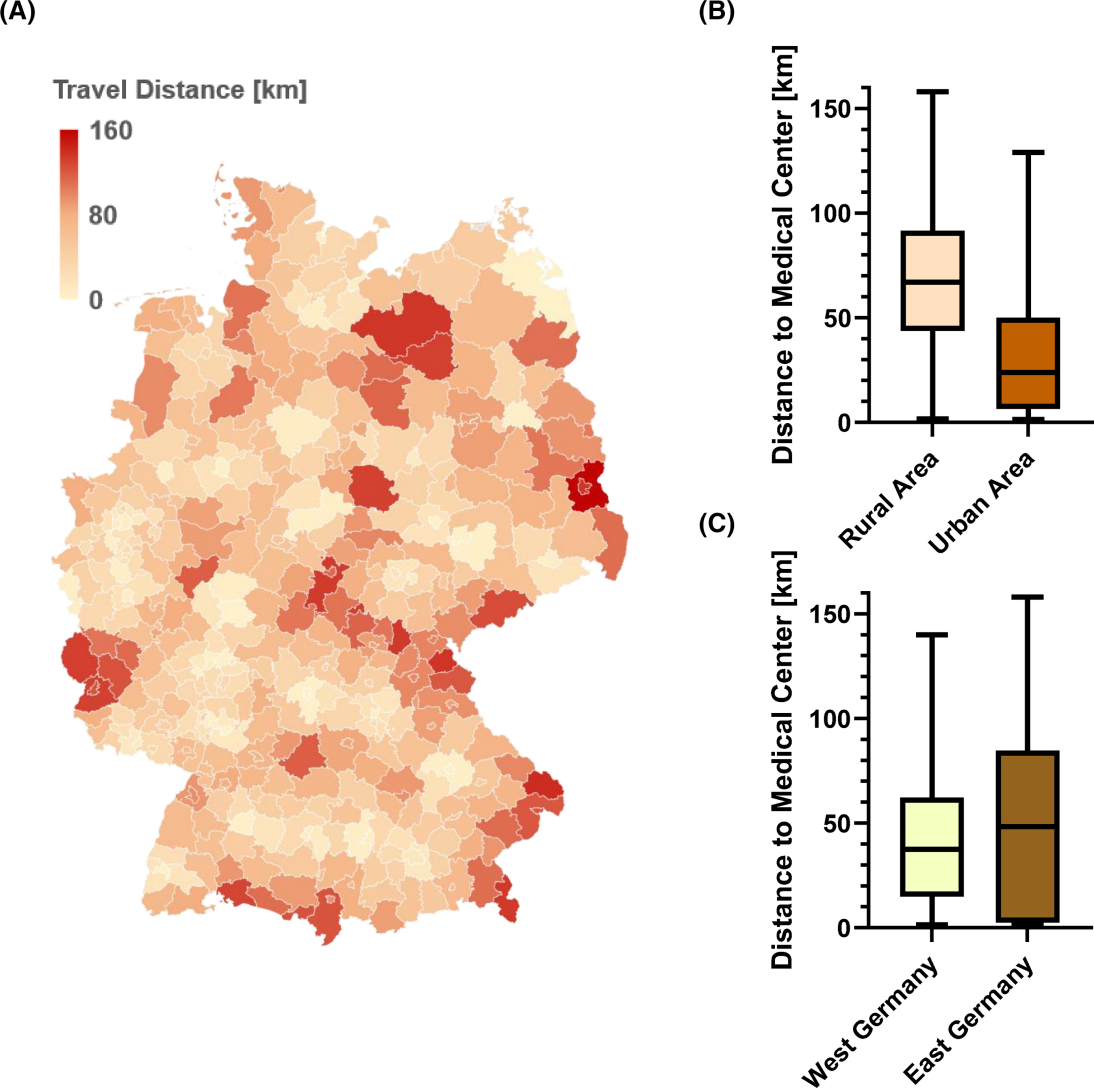
Estimated mean travel distance to the nearest university hospital. (A) The mean travel distance from each German district to the nearest University hospital is shown. Travel distances between a district (center point) and the nearest university hospital (38 in total) were estimated by calculation the distances (km) in google maps. (B) Travel distances with among rural and urban areas as well as between (C) West and East Germany are presented with box and whiskers plots. Medians, upper and lower quartiles are shown as boxes and minima/maxima as whiskers.

Estimated mean travel distances to medical centers (university hospitals) vary by area of residence in Germany (Figure 2A). On average, there were 68.6 versus 66.1 general physicians, 6.2 versus 5.4 ENT physicians, and 659.2 versus 628.5 hospital beds per 100,000 inhabitants counted in the year 2021 in East and West Germany, respectively (*p* < 0.0001). We observed a difference in the mean estimated travel distances to the next large university medical center as a proxy for specialized cancer centers (Figure 2B,C) between rural and urban areas (69.4 km [SD = 32.7] vs. 30.6 km [SD = 28.6]; *p* < 0.0001) as well as between East and West Germany (51.1 km [SD = 42.9] vs. 42.5 [SD = 28.5]; *p* < 0.0001).

### 3.3 Mean age of HNCP rises and gender imbalance equilibrates in both, rural and urban districts

**FIGURE 3:**
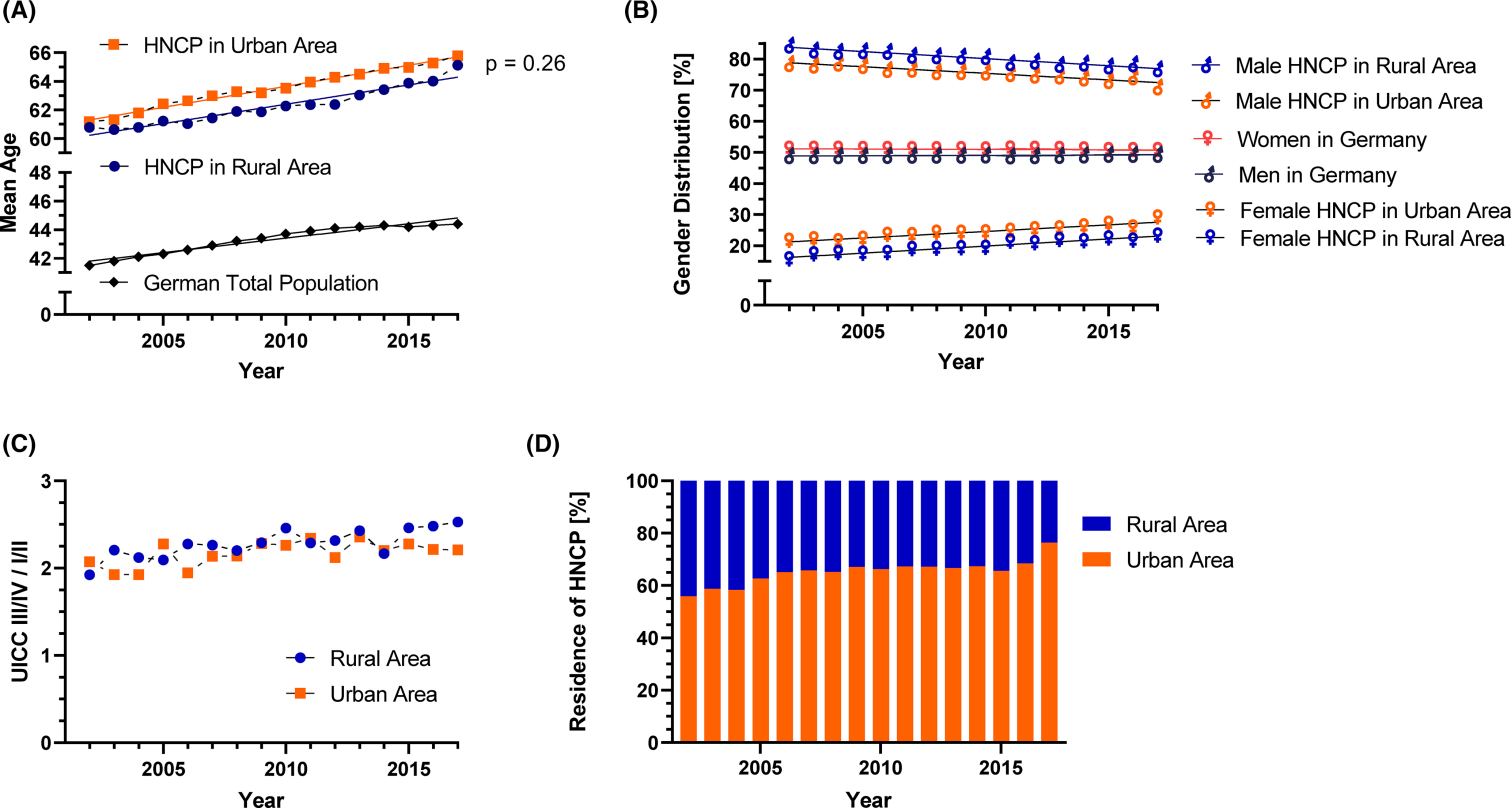
Development of age, gender ratio, stage, and residence. (A) Mean age the head and neck cancer patients (HNCP) by area in comparison to the German population is shown over time with trendlines. (B) Relative fraction for gender distribution by area is shown in comparison to the gender distribution in the general German population, accompanied by trendlines. (C) The relation of advanced to low stage and (D) allocation of HNCP in rural and urban districts is displayed during the observation period.

Mean age of HNCP in both rural areas and urban areas is rising significantly (*p* < 0.001; Figure 3A) maintaining the small age gap and increasing at a comparable rate (slope coefficients do not differ significantly: rural area: 0.29, urban area: 0.27; *p* = 0.26). Consequently, mean age increased in rural areas from 60.8 in 2002 to 65.1 years in 2017 and in urban areas from to 61.2 to 65.7 years, respectively. At the same time the mean age of the German population rose from 41.5 to 44.4 years. The gender imbalance, resulting from higher numbers of male HNCP, was more pronounced in rural areas. The mean ratio of men to women in rural versus urban areas is 4.1 and 3.1 respectively (*p* < 0.0001). However, this ratio seems to decrease (Figure 3B) over the observation period at a comparable rate in both rural and urban areas (slope coefficient of men portion in rural areas: −0.45, slope coefficient of men portion in urban areas: −0.42; *p* = 0.46). In addition, the slope coefficients of the portion of men and women in the general population in Germany do not differ (men: 0.02; women: −0.02; *p* = 0.46). During the time period of 2002–2017 there was no change in the ratio of advanced to low UICC stage (Figure 3C). In fact, the slope coefficients for rural and urban areas are close to zero (0.01, 0.00; *p* = 0.26).

### 3.4 Increasing predominance of HNCP in urban areas

The proportion of HNCP living in urban areas is significantly higher than those living in rural areas (*p* < 0.0001). Over time, this difference seemed to grow (Figure 3D). The proportion of HNCP living in urban areas increased from 55.9% in the year 2002 to 76.4% in the year 2017. However, it is possible that the increase of HNCP in urban areas is overestimated because of reporting delays after 2015.

### 3.5 Residence in rural areas is a negative prognostic factor

**FIGURE 4:**
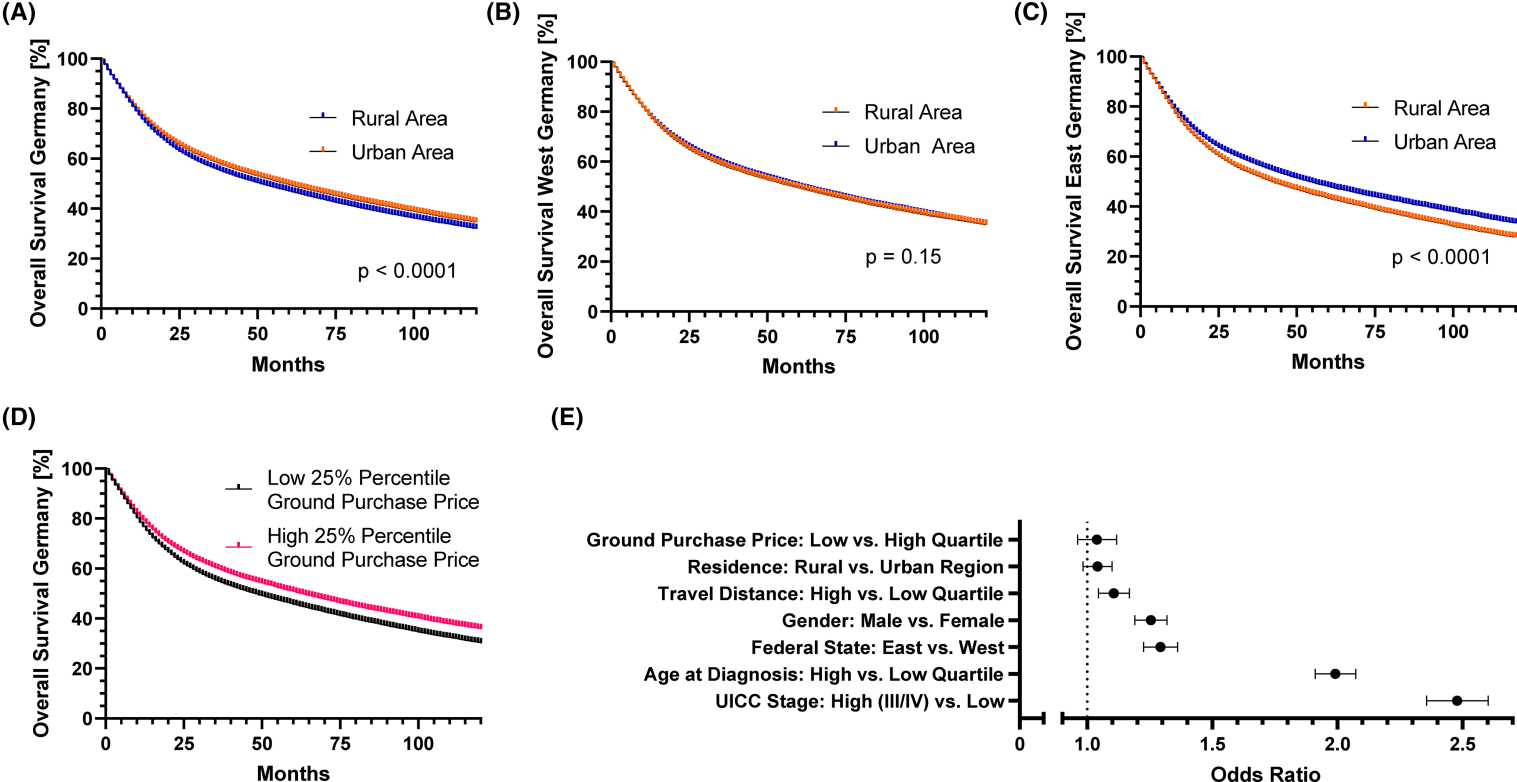
Survival of HNCP by area of residence. (A) The Survival of all HNCP in relation to their residence is displayed. (B) The Survival of HNCP living in West Germany and (C) East Germany is displayed. (D) Survival of HNCP is graphed in relation to the lower and upper quartile of mean ground purchase price in the area of residence in the year 2011 (E) Forrest plot showing hazard ratios from a multivariate analysis of risk factors for succumbing to cancer (E).

Looking at the median overall survival of HNCP there was a small, but significant difference in survival in favor of HNCP from urban districts compared to rural areas (Figure 4A). The median overall survival was 51 months (SEM = 0.6; CI: 49.9–52.1) in rural and 59 months (SEM = 0.5; CI: 58.1–59.9) in urban areas (*p* < 0.0001). However, when subdivided into urban and rural areas in West (Figure 4B) and East (Figure 4C) Germany, this survival disadvantage for rural areas was not present in West Germany (median survival: 59 months [SEM = 0.8; CI: 57.4–60.6] in rural areas vs. 60 months [SEM = 0.5; CI: 59.0–61.0] in urban areas; *p* = 0.15). Instead, the survival disadvantage for rural compared to urban areas was driven by East Germany (median survival: 42 months [SEM = 0.7; CI: 40.5–43.5] in rural areas vs. 54 months [SEM = 1.2; CI: 51.7–56.3] in urban areas; *p* < 0.0001).

### 3.6 Socioeconomic status seems to affect survival of HNCP in Germany

Finally, mean ground purchase prices from 2011 were chosen as a surrogate marker for socioeconomic status, because land values account for a wide variety of socioeconomic and location factors reflecting the purchasing power of residents. Mean ground purchase price within the lowest quartile was ≤41.04€ versus ≥152.71€ in the highest quartile. The lowest prices were found in rural areas in East Germany. The categorization of the ground purchasing price performed in this way highly correlated with the division of the federal states into East and West (r = 0.58). Median overall survival within the bottom quartile was 48 versus 63 months in the top quartile (Figure 4D; *p* < 0.0001). To test for independence of the demographic factors analyzed, a multivariate analysis including all parameters analyzed was performed (Figure 4E). Unsurprisingly, we saw that high‐tumor stage (UICC III, IV vs. UICC I, II) and age (top quartile [≥70.3 years] versus bottom quartile [≤54.9 years]) were the most important and independent risk factors (*B* = 2.48, *B* = 1.99; *p* < 0.0001). These risk factors were then interestingly followed by federal states (East vs. West), gender (male vs. female), and travel distance (top [≥66.7 km] versus bottom [≤11 km] quartile) with *B* = 1.29, *B* = 1.25 and *B* = 1.11, respectively (*p* < 0.0001). But looking at spatial type of residence (rural vs. urban) and ground value (bottom vs. top quartile) we only observed nonsignificant hazard ratios of B = 1.04 and *B* = 1.04 (*p* = 0.18; *p* = 0.35).

## 4 DISCUSSION

In this nationwide cancer registry study with inclusion of 212,920 HNC patients we observed differences in age, gender distribution, estimated travel distance, socioeconomic status, infrastructure, HNC incidence, and stage among rural and urban inhabitants between 2002 and 2017. There were also similar demographic development trends between rural and urban areas like a rising mean age and the trend to equilibration of gender imbalance. We found a disparity in survival outcomes of patients in rural areas compared to urban areas in Germany, primarily because of a survival disadvantage for HNCP from rural areas in East Germany. Thus, the factor “East versus West” outperforms “rural versus urban.” However, due to the differences between ground values and travel distances between East and West Germany, the categorization into East and West already accounts for these factors. Notably, in contrary to the availability of specialized care centers, the availability of primary medical care in the East is higher than in the West, emphasizing the importance of specialized care for more complex diseases and socioeconomic status. Of Course, measures for primary and secondary prevention of HNC by primary medical care are indispensable in the proximity of the patient, even though they are not as well established in the broad population yet as they may be for cardiovascular diseases.^14^

Primary disease prevention strategies need to be tailored to these demographic features for urban and rural areas. As smoking and alcohol abuse are major risk factors for HNC, different substance abuse patterns may be present in rural and urban areas and definitely are in East and West Germany to the disadvantage of the East.^15^ In 2019, 26.9% of men and 19.1% of women smoked daily^16^—of note, the prevalence of smokers was higher in German cities than in rural areas.^17^ Additionally, substance abuse negatively correlates with education and wealth in Germany.^18^ However, there were no reliable quantitative data available for pack years of smoking history and the amount of alcohol consumption in standard drinks or grams of alcohol. To navigate primary prevention efforts, such data would be very helpful. Documentation and availability of substance abuse data in HNCP may be used to guide anti‐smoking and anti‐alcohol campaigns. Another important risk factor for the development of oropharyngeal carcinomas is the human papillomavirus (HPV). Increasing prevalence of between 11.5 and 55% have been reported in Germany, a situation that is to be countered using appropriate vaccines.^19^

One more contributing factor may be quality of care. Treatment at specialized HNC centers (HNCC) has been shown to result in a longer survival.^2^ The first three HNCC certified by *Deutsche Krebsgesellschaft* were established in 2010. Today (March/2023) there are 78 certified HNCC in Germany. Especially in rural areas of East Germany, reducing travel distance to specialized care centers may be a promising strategy for secondary prevention. Cancer registries are currently not documenting whether patients were treated at a HNCC or not. There is a political discussion whether treatment at HNCC should be mandatory. On the one hand, this may improve quality and standardization of treatment, but on the other hand, longer travel to receive care may result in a higher level of stress and higher cost for patients. In many European countries such as the Netherlands or Denmark, treatment at specialized cancer centers is mandatory. This centralization results in a high consistency and a high rate of participation in clinical trials further improving patient care. At the same time, patients must endure longer journeys during treatment.^20^

There seems to be a high level of international interest in the relationship between treatment in rural or urban areas and survival/quality of life of cancer patients. An analysis including 583 cancer survivors from the Irish National Cancer Database^21^ as well as an analysis of 261 American HNCP^22^ showed that HNCP in rural areas are more likely to report about decreased quality of life concerning physical and emotional health status. Moreover, a recent American study enclosing 146,256 HNCP between 2004 and 2015 reported that the median survival of Black HNCP living in rural areas was worst (43.1 months) compared to Black HNCP in urban areas as well as in comparison to white HNCP. White HNCP living in urban areas had the best median survival (67.0 months) in the study. The authors conclude that in the United States of America an important factor of disparity in survival between rural and urban areas is race. As a possible contributing factor, the authors discussed a stronger distrust in health care by Black HNCP (discrimination, misinformation), underinsurance, lower income, and lack of medical knowledge. Further on, they emphasize that rural inhabitants have less access to tertiary care centers, medical professionals, and public transport. Additionally, people living in those rural communities have a higher prevalence of cigarette smoking.^6^ Whereas we could not analyze the factor race in our cohort, other observations, and interpretations of contributing factors such as disparity of income, education and higher distrust in state institutions may be a commonality. These results are in a line with studies from other countries like Egypt^5^ or Canada.^23^ On the other hand, no spatial‐specific differences in HNCP survival were observed in Britain^7^ or in our case in West Germany. This could be due to a downturn of socioeconomic deprivation in combination with low travel burden in the latter countries. It may therefore be considered that living in a rural area is not a risk factor in general, if a stable socioeconomic structure, prevention measures and access to health care is facilitated. Adaptations in the health care system are a balancing act between centralization of health care and sufficient accessibility for everyone.^20^ In Germany, aside from spatial differences in rural/urban areas, at least small disparity between East and West Germany persists more than 30 years after reunification. Since then, previously published data described shrinking differences in overall survival amounting to 0.4 years for women and 1.5 years for men in 2002 (largely accounting to differences in cardiovascular mortality).^24^ There are a number of contributing factors to this phenomenon such as longer travel distance to agglomeration zones, higher rates of emigration, a lower degree of academization, lower employment rates, lower income, and a smaller level of facilities for public services and infrastructure in East Germany.^25^ Nonetheless, conditions seem to have currently improved, so that life expectancy in East and West Germany is now almost the same.^1^

In the future, a shrinkage of the population, aging, and individual isolation may additionally threat interregional balance.^26^ Such a shift in population structure (age, gender, nationality, habits, settlement patterns, etc.) may subsequently influence HNCP survival.^27^ Specific health care adaptions and support systems are needed. Such support may be of monetary nature by supporting economically weak areas and improving required infrastructure.^28^, ^29^ Also, in order to optimize cancer data analysis, documentation of cancer data should be expanded, accelerated, and standardized in Germany. Simplified accessibility of timely data for research purposes should be discussed and weighted against data protection concerns.

Our study may be limited by the quality and extent of accessible data. Individual patient data of socioeconomic status, details on substance abuse history, comorbidity, and education status were not available. There is also a significant documentation delay and a high rate of incomplete data in the cancer registry datasets.

## 5 CONCLUSIONS

This nationwide study revealed differences in survival among rural and urban HNCP primarily in East Germany at the expense of HNCP from rural areas. This disparity seems to be driven primarily by socioeconomic factors.

## AUTHOR CONTRIBUTIONS

**Julius M. Vahl:** Conceptualization (supporting); data curation (lead); formal analysis (equal); funding acquisition (supporting); investigation (equal); methodology (equal); project administration (supporting); resources (supporting); software (lead); supervision (supporting); validation (equal); visualization (lead); writing – original draft (lead); writing – review and editing (equal). **Gabriele Nagel:** Data curation (supporting); formal analysis (supporting); supervision (supporting); validation (supporting); writing – review and editing (supporting). **Ayla Grages:** Validation (supporting); writing – review and editing (supporting). **Matthias Brand:** Validation (supporting). **Adrian von Witzleben:** Validation (supporting). **Michael Sonntag:** Validation (supporting). **Marie‐Nicole Theodoraki:** Validation (supporting); writing – review and editing (supporting). **Jens Greve:** Validation (supporting); writing – review and editing (supporting). **Tsima Aboukors:** Validation (supporting). **Michael Denkinger:** Validation (supporting); writing – review and editing (supporting). **Dhayana Dallmeier:** Validation (supporting). **Christian Idel:** Validation (supporting); writing – review and editing (supporting). **Thomas K. Hoffmann:** Project administration (supporting); resources (supporting); validation (supporting); writing – review and editing (supporting). **Simon Laban:** Conceptualization (lead); data curation (supporting); formal analysis (equal); funding acquisition (lead); investigation (equal); methodology (equal); project administration (lead); resources (lead); software (supporting); supervision (lead); validation (equal); visualization (supporting); writing – original draft (supporting); writing – review and editing (equal).

## FUNDING INFORMATION

No funding was received.

## CONFLICT OF INTEREST STATEMENT

Simon Laban: Advisory Boards: Merck Sharp & Dohme (M.S.D.), Bristol Myers, Squibb (B.M.S.), Astra Zeneca (A.Z.). Honoraria: M.S.D., B.M.S., A.Z., Merck Serono. Thomas K. Hoffmann: Advisory Boards: M.S.D., B.M.S. Honoraria: M.S.D., B.M.S., Merck Serono. All other authors declared no conflict of interests in conjunction with this work.

## ETHICS STATEMENT

No ethic vote is needed in agreement with our ethics committee because data received were anonymized and patient‐related data concerning date of birth, diagnosis, and death were also anonymized (written confirmation available [OCT/2022]).

## INFORMED CONSENT

Epidemiological cancer registration in Germany is regulated by state laws and data received were anonymized facilized. The Federal Cancer Registry Data Act of 2009 defines the tasks of the Center for Cancer Registry Data at the Robert Koch Institute as the national evaluation center. This means that all physicians and dentists in the state are required to report cancer cases they are involved in diagnosing, treating or following up to the state cancer registry. Patients consent is not required for this.

## Supporting information


Figure S1

## Data Availability

The original dataset cannot be shared. The data on HNC patients in Germany have been requested in a multistep process from the German cancer registry for a dedicated purpose and specific group of persons only and must not be shared. The data on the general population, which were ordered in different context from de federal statistical office and suboffices at a charge, may only be used applicant‐related too. The hereby used data of the German federal Institute for building, urban affairs and spatial development is publicly accessible.

## References

[1] Lampert T , Müters S , Kuntz B , Dahm S , Nowossadeck E . 30 years after the fall of the Berlin Wall: regional health differences in Germany. J Health Monit. 2019;4(suppl 2):2‐23. 10.25646/6077 PMC8832371 35586335

[2] Schoffer O , Klinkhammer‐Schalke M , Schmitt J . Überlebensvorteile bei Behandlung in zertifizierten Krebszentren. Gesundheit Und Gesellschaft Wissenschaft. 2022;Heft 4:7‐15.

[3] Nasser M , Sawicki P . Institute for Quality and Efficiency in Health Care. Commonwealth Fund; 2009. 19639711

[4] Bau‐ S‐uR, Bundesinstitut . Stadt‐Und Raumforschung, 2010: Laufende Raumbeobachtung–Raumabgrenzungen. 2022. https://www.bbsr.bund.de/BBSR/DE/forschung/raumbeobachtung/Raumabgrenzungen/deutschland/gemeinden/Raumtypen2010_vbg/Raumtypen2010_alt.html

[5] Attar E , Dey S , Hablas A , et al. Head and neck cancer in a developing country: a population‐based perspective across 8 years. Oral Oncol. 2010;46(8):591‐596. 20619719 10.1016/j.oraloncology.2010.05.002 PMC3223856

[6] Clarke JA , Despotis AM , Ramirez RJ , Zevallos JP , Mazul AL . Head and neck cancer survival disparities by race and rural–urban contextHNC rural–urban survival disparities by race. Cancer Epidemiol Biomarkers Prev. 2020;29(10):1955‐1961. 32727721 10.1158/1055-9965.EPI-20-0376 PMC9073403

[7] Kim JD , Firouzbakht A , Ruan JY , et al. Urban and rural differences in outcomes of head and neck cancer. Laryngoscope. 2018;128(4):852‐858. 28940575 10.1002/lary.26836

[8] Nennecke A , Geiss K , Hentschel S , et al. Survival of cancer patients in urban and rural areas of Germany—a comparison. Cancer Epidemiol. 2014;38(3):259‐265. 24680643 10.1016/j.canep.2014.02.011

[9] Vahl JM , Wigand MC , Denkinger M , et al. Increasing mean age of head and neck cancer patients at a German tertiary referral center. Cancer. 2021;13(4):832. 10.3390/cancers13040832 PMC7922863 33671152

[10] Vahl J , Von Witzleben A , Welke C , et al. Influence of travel burden on tumor classification and survival of head and neck cancer patients. Eur Arch Otorhinolaryngol. 2021;278(11):4535‐4543. 33877433 10.1007/s00405-021-06816-3

[11] Vahl JM . Regional Outcome Disparities in German Head and Neck Cancer Patients: Shorter Survival in Eastern Germany. 2023. 10.1002/cam4.6690 PMC10726835 38037808

[12] Zentrum für Krebsregisterdaten (ZfKD) im Robert Koch‐Institut . Datensatz des ZfKD auf Basis der epidemiologischen Landeskrebsregisterdaten, verfügbare Diagnosejahre bis 2017. 2020.

[13] für Justiz B . Gesetz über die Grundqualifikation und die Weiterbildung der Fahrer bestimmter Kraftfahrzeuge für den Güter‐ oder Personenkraftverkehr: Bundesministerium der Justiz. 2022. https://www.gesetze‐im‐internet.de/bkrfqg_2020/BJNR257510020.html

[14] Di Girolamo C , Nusselder WJ , Bopp M , et al. Progress in reducing inequalities in cardiovascular disease mortality in Europe. Heart. 2020;106(1):40‐49. 31439656 10.1136/heartjnl-2019-315129 PMC6952836

[15] Atzendorf J , Apfelbacher C , de Matos EG , et al. Do smoking, nutrition, alcohol use, and physical activity vary between regions in Germany? Results of a cross‐sectional study. BMC Public Health. 2020;20:1‐8. 32164635 10.1186/s12889-020-8352-2 PMC7068923

[16] Eurostat Data Browser Statistics|Eurostat (europa.eu). https://ec.europa.eu/eurostat/databrowser/view/HLTH_EHIS_SK1E__custom_1721096/bookmark/table?lang=en&bookmarkId=602df4da‐f30d‐4acc‐b509‐d5992435d3f6&page=time:2019

[17] Völzke H , Neuhauser H , Moebus S , et al. Urban‐rural disparities in smoking behaviour in Germany. BMC Public Health. 2006;6(1):1‐8. 16756650 10.1186/1471-2458-6-146 PMC1513566

[18] Moor I , Winter K , Rathmann K , Ravens‐Sieberer U , Richter M . Alkohol‐, tabak‐und cannabiskonsum im jugendalter–querschnittergebnisse der HBSC‐Studie 2017/18. 2020.

[19] Reuschenbach M , Tinhofer I , Wittekindt C , Wagner S , Klussmann JP . A systematic review of the HPV‐attributable fraction of oropharyngeal squamous cell carcinomas in Germany. Cancer Med. 2019;8(4):1908‐1918. 30821126 10.1002/cam4.2039 PMC6488137

[20] Vahl JM , Böhm F , Brand M , von Witzleben A , Hoffmann TK , Laban S . Zentralisierung, Spezialisierung und Ambulantisierung der Versorgung von Kopf‐Hals‐Tumorpatienten. Laryngorhinootologie. 2022;101(12):987‐991. 35675834 10.1055/a-1851-5257

[21] Thomas AA , Timmons A , Molcho M , et al. Quality of life in urban and rural settings: a study of head and neck cancer survivors. Oral Oncol. 2014;50(7):676‐682. 24731737 10.1016/j.oraloncology.2014.03.007

[22] Adamowicz JL , Christensen A , Howren MB , et al. Health‐related quality of life in head and neck cancer survivors: evaluating the rural disadvantage. J Rural Health. 2022;38(1):54‐62. 33720456 10.1111/jrh.12571 PMC8477149

[23] Walker BB , Schuurman N , Auluck A , Lear SA , Rosin M . Socioeconomic disparities in head and neck cancer patients' access to cancer treatment centers. Rural Remote Health. 2017;17(3):1‐11. 10.22605/RRH4210 28870083

[24] Razum O , Altenhöner T , Breckenkamp J , Voigtländer S . Social epidemiology after the German reunification: east vs. west or poor vs. rich? Int J Public Health. 2008;53(1):13‐22. 18522365 10.1007/s00038-007-6116-8

[25] Schrader H . Impact assessment of the EU structural funds to support regional economic development in rural areas of Germany. J Rural Stud. 1994;10(4):357‐365.

[26] Hullen G . Bevölkerungsentwicklung in Deutschland. In: Frevel B , ed. Herausforderung demografischer Wandel. VS Verlag für Sozialwissenschaften; 2004:15‐25.

[27] Faye‐Lund H , Abdelnoor M . Prognostic factors of survival in a cohort of head and neck cancer patients in Oslo. Eur J Cancer B Oral Oncol. 1996;32(2):83‐90. 10.1016/0964-1955(95)00073-9 8736169

[28] Erbe S . Hat sich der Risikostrukturausgleich in der GKV bewährt? Wirtschaftsdienst. 2006;86(5):333‐340.

[29] Hallett AJH , Ma Y . East Germany, West Germany, and their Mezzogiorno problem: a parable for European economic integration. Econ J. 1993;103(417):416‐428.

