# Supplementary figures and images for: Demographics and access to head and neck cancer care in rural areas compared to urban areas in Germany
Source: Cancer Med. 2023 Sep 14;12(18):18826–36. doi: 10.1002/cam4.6505 (PMC10557897; doi:10.1002/cam4.6505)

A

Portion of German HNCP reports by  
each district [%]

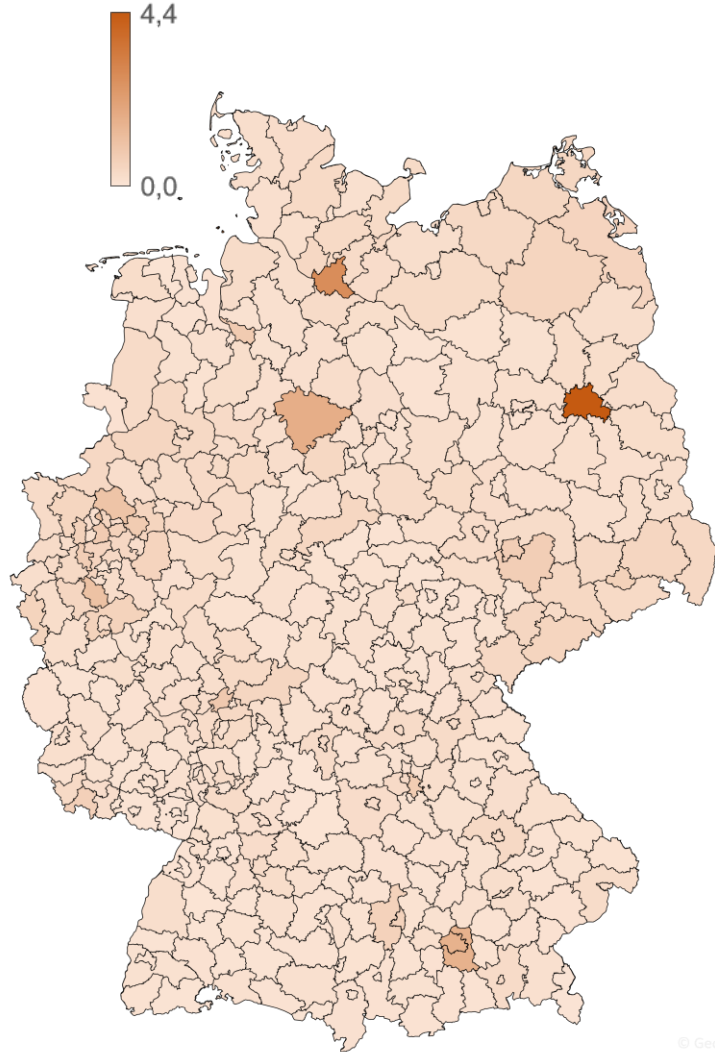

B

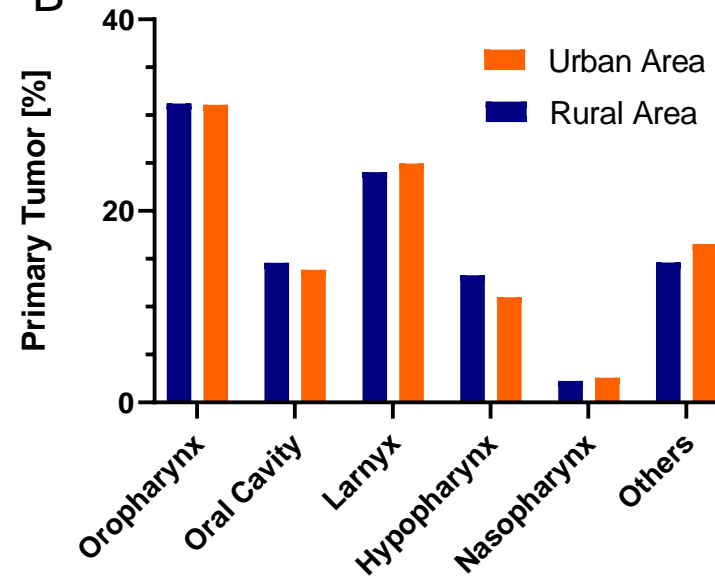

C

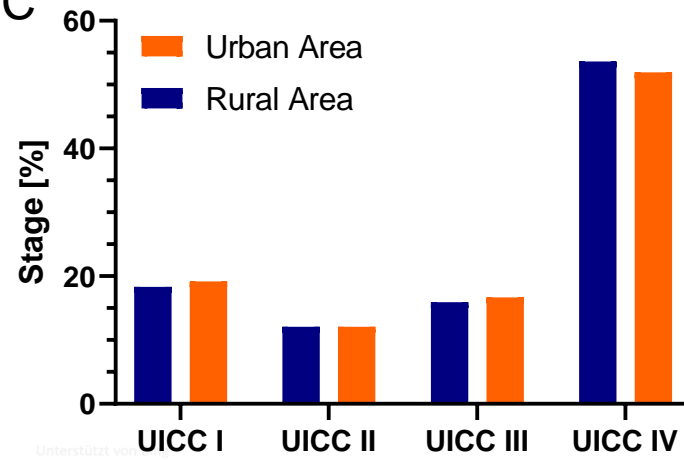

Supplement: Supplementary file 1 — Figure S1 [file CAM4-12-18826-s001.pdf]
